# Periodontitis Prevalence in a West African Sub-Population Using Two Definitions: A Cross-Sectional Study in Ouagadougou (Burkina Faso)

**DOI:** 10.3290/j.ohpd.c_2520

**Published:** 2026-03-04

**Authors:** Abdoulaziz Diarra, Jean Claude Romaric Pingdwindé Ouédraogo, Estelle Flore Bandré, Olivier Huck, Estelle Noëla Hoho Youl, Kevimy Agossa

**Affiliations:** a Abdoulaziz Diarra Assistant Professor, Periodontology, Training and Research Unit in Health Sciences (UFR/SDS) University of Ouaga I, Pr. Joseph Ki-Zerbo, Ouagadougou, Burkina Faso. Conception, study design, data collection, data interpretation, wrote the manuscript.; b Jean Claude Romaric Pingdwindé Ouédraogo Medical Doctor, Institut de Recherche en Sciences de la Santé (IRSS/CNRST), 03 PB 7047, Ouagadougou 03, Ouagadougou, Burkina Faso. Statistical analysis or interpretation of the data, read and approved the manuscript.; c Estelle Flore Bandré Doctor of Dental Surgery, Periodontology, Training and Research Unit in Health Sciences (UFR/SDS), University of Ouaga I, Pr. Joseph Ki-Zerbo, Ouagadougou, Burkina Faso. Acquisition, analysis of the data, read and approved the manuscript.; d Olivier Huck Professor of Periodontology, University of Strasbourg, Faculty of Dentistry Robert Frank, INSERM, UMR 1260, Regenerative Nanomedicine, Strasbourg, France. Critical proofreading and discussion, read and approved the manuscript.; e Estelle Noëla Hoho Youl Professor of Pharmacology, Training and Research Unit in Health Sciences (UFR/SDS), University of Ouaga I, Pr. Joseph Ki-Zerbo, Ouagadougou, Burkina Faso. Critical proofreading and discussion, funding acquisition, read and approved the manuscript.; f Kevimy Agossa Professor of Periodontology, Univ. Lille, CHU Lille, INSERM, Department of Periodontology School of Dentistry, U1008 Advanced Drug Delivery Systems, Lille, France; Department of Periodontics and Oral Medicine, University of Michigan School of Dentistry, Ann Arbor, MI, USA. Conception, critical proofreading and discussion, read and approved the manuscript.

**Keywords:** Africa, epidemiology, periodontitis, population surveillance, risk factors.

## Abstract

**Purpose:**

This study evaluated prevalence, severity and risk indicators of periodontitis among urban West African adults using CDC/AAP 2012 and EFP/AAP 2018 criteria.

**Materials and Methods:**

A cross-sectional study among adults attending four public dental centers in Ouagadougou, Burkina Faso was conducted. Sociodemographic and risk factors were collected by interview, and full-mouth periodontal exams were performed at six sites per tooth. Both CDC/AAP 2012 and EFP/AAP 2018 definitions were applied. The performance of the EFP/AAP criteria was compared with CDC/AAP as the reference.

**Results:**

A total of 749 participants (mean age: 34.32 ± 11.51 years; 52.34% female) were included. The prevalence of periodontitis was 71.83% using EFP/AAP criteria (stage I: 16.02%; stage II: 24.57%; stage III/IV: 31.25%) and 56.21% with CDC/AAP (mild: 6.28%; moderate: 38.45%; severe: 11.48%). Compared with CDC/AAP, EFP/AAP showed high sensitivity (99.29%) but low specificity (28.96%), with a positive predictive value of 64.21% and negative predictive value of 96.94%. Increasing age (CDC/AAP: OR = 1.03, 95% CI: 1.01–1.05, p = 0.001 | EFP/AAP: OR = 1.03, 95% CI: 1.00–1.07, p = 0.040) and smoking (CDC/AAP: OR = 2.78, 95% CI: 1.12–6.89, p = 0.028 | EFP/AAP: OR = 2.24, 95% CI: 1.03–4.86, p = 0.040) were consistently associated with higher odds of periodontitis.

**Conclusion:**

Periodontitis was highly prevalent in this urban West African population, with advanced disease affecting up to one-third of adults. The EFP/AAP classification identified more cases but showed lower specificity, potentially overestimating disease burden in settings with high prevalence but predominantly moderate disease.

Periodontitis is a chronic immune-inflammatory disease initiated by a dysbiotic biofilm, resulting in progressive destruction of the tooth-supporting structures.^52^ It represents a major global public health burden, with significant implications for both individuals and healthcare systems.^33,36,53,58^ Severe periodontitis affects over one billion people worldwide^46^ and remains a leading cause of tooth loss in adults, leading to impaired mastication, nutritional deficiencies, esthetic concerns, and reduced quality of life.^20,33,38,60^ Beyond the oral cavity, periodontitis is strongly associated with systemic conditions such as diabetes mellitus, cardiovascular diseases, and over 50 other non-communicable diseases.^7,10,43,54^ It has recently been proposed that periodontitis should be regarded as a systemic disease in its own right, owing to its measurable impact on systemic health and its bidirectional interactions with multiple chronic comorbidities.^61^ The economic impact is substantial, with direct and indirect costs estimated at $154 billion in the United States and €159 billion in Europe, in 2018.^6^

The global distribution of periodontitis varies greatly across geographic regions, reflecting differences in individual risk profiles, environmental exposures, and healthcare system determinants.^3,21,33,36,37,48^ Persistent socioeconomic and structural inequities further drive pronounced disparities in oral health outcomes.^21^ Recent estimates indicate that sub-Saharan Africa and South Asia bear a disproportionate burden of disease.^12,21,36,63^ However, true prevalence levels remain uncertain because of substantial methodological heterogeneity, limited surveillance capacity, and the use of diagnostic tools that insufficiently capture clinical severity. The Community Periodontal Index (CPI/CPITN)—commonly applied in earlier surveys—fails to adequately detect attachment loss or classify disease stages.^25,26,30,32^ Moreover, pronounced disparities in income, education, and access to care across the continent may further bias aggregate estimates.^45^

Between 2000 and 2020, the urban population across West African countries increased from 82 million to approximately 200 million, making it one of the most rapidly urbanizing regions globally.^49^ Urbanization has been proposed as a key driver of the growing burden of chronic diseases in low- and middle-income countries, through increased exposure to environmental and lifestyle factors such as poor diet, sedentary behavior, alcohol and tobacco use, and psychosocial stress,^27,34,40,42,55^ all established or potential risk factors for periodontitis.^16,22^

Burkina Faso (BF), a landlocked West African country with over 23 million inhabitants, illustrates healthcare challenges typical of low-income settings, including limited epidemiological surveillance and restricted access to dental care.^1^ Ouagadougou, the capital, is home to more than 10% of the national population and presents considerable ethnic and socioeconomic diversity, making it a suitable setting for updated epidemiological data exploring oral, general health, lifestyle, and socioeconomic determinants.^47^ The most recent oral health data from Burkina Faso, collected in 2014, used the Community Periodontal Index (CPI) to assess periodontal status in rural populations (21% of adolescents and 61% of adults had an attachment loss ≥4 mm);^13^ however, no data are available for urban settings.

This study aimed to assess the prevalence, severity, and risk indicators of periodontitis in a hospital-based adult population in Ouagadougou using both the CDC/AAP 2012 definition^18^ and the EFP/AAP 2018 classification.^52^ A further objective was to generate updated epidemiological data to support public health decision-making and to examine how these diagnostic criteria influence disease estimates.

## MATERIALS AND METHODS

### Study Design and Settings 

This hospital-based cross-sectional study was conducted from March 2023 to August 2023 in Ouagadougou (estimated population: 2,415,266), located in Burkina Faso, West Africa.^47^ Located in the central province of Kadiogo, Ouagadougou serves as the country’s administrative, economic, and cultural hub and hosts the most extensive and accessible public dental infrastructure. The study was conducted at the four existing public dental facilities in the city: University Hospital Yalgado Ouédraogo (CHUYO), University Hospital of Bogodogo (CHUB), University Hospital of Tengandogo (CHUT), and the Municipal Oral Health Center of Ouagadougou (CMSBD).

### Study Population 

All individuals aged 18 years or older who presented for outpatient dental consultation during the study period and resided in Ouagadougou or its surroundings were eligible. Inclusion required participants to be in the adult permanent dentition phase with ≥16 teeth (excluding third molars). Exclusion criteria included pregnancy, medical contraindications to periodontal probing, and periodontal or antibiotic treatment within the three months preceding data collection. Written informed consent was obtained from all participants prior to enrolment.

### Sample Size and Sampling 

In the absence of prior prevalence data for periodontitis in this population, a theoretical prevalence of 50% was assumed, in line with global adult estimates.^28^ The required sample size was calculated using Cochran’s formula:^14^


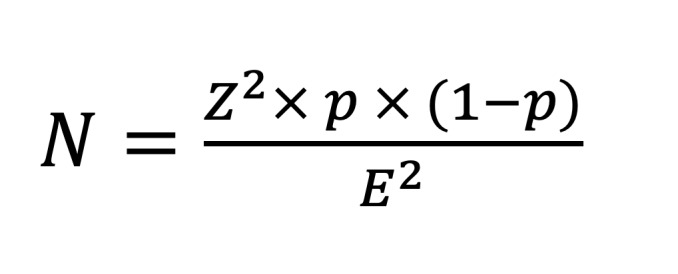


where Z = 1.96 (for 95% confidence), p = 0.5, and E = 0.05. The minimum required sample size was estimated at 384 participants. To increase statistical power and support subgroup analyses, all eligible individuals attending consultations during the study period were included. A total of 749 participants were ultimately enrolled.

### Data Collection and Variables

The following data were collected through a structured face-to-face interview, followed by a clinical oral examination.

Sociodemographic characteristics: age, sex, marital status, education, occupation, residence, ethnicity, religion, health insurance
Medical history: self-reported comorbidities (e.g., diabetes, hypertension, cardiovascular disease)
Dental history: frequency and timing of dental visits, main reason for last visit, previous periodontal treatment, tooth loss
Oral hygiene practices: frequency and tools used for dental and interdental cleaning, professional cleaning history
Lifestyle factors: tobacco and alcohol use, fruit and vegetable intake

The primary outcome was periodontitis, assessed for presence, extent, and severity using the CDC/AAP 2012 and EFP/AAP 2018 classifications.^18,52,57^ All other variables were analyzed as potential explanatory or confounding factors.

### Clinical Examination

Clinical periodontal examinations were conducted in dental offices using standardized protocols. Four trained examiners performed all clinical assessments, each assisted by a dental assistant or dental student.

A sterile examination kit—including a plane dental mirror, tweezers, and a Williams periodontal probe (Hu-Friedy; Chicago, IL, USA)—was used. All fully erupted teeth, excluding third molars, were examined at six sites per tooth.^11^

The following clinical parameters were recorded:

Plaque index (PI): assessed using the O’Leary method, and expressed as the percentage of tooth surfaces with dental plaque.^50^
Bleeding on probing (BOP): defined as the presence of gingival bleeding within 20 s of gentle probing,^2^
Gingival recession (GR): distance from the cementoenamel junction (CEJ) to the gingival margin.^24^
Probing pocket depth (PPD): distance from the free gingival margin (FGM) to the base of the sulcus/pocket.^62^
Clinical attachment level (CAL): calculated as the sum of GR and PPD.^5^

### Periodontitis Case Definitions

Periodontitis was classified using two internationally recognized systems, as follows.

#### CDC/AAP 2012 case definition^18^

Mild periodontitis: ≥2 interproximal sites with CAL ≥3 mm and ≥2 interproximal sites with PPD ≥4 mm (not on the same tooth), or one site with PPD ≥5 mm.
Moderate periodontitis: ≥2 interproximal sites with CAL ≥4 mm or ≥2 interproximal sites with PPD ≥5 mm (not on the same tooth).
Severe periodontitis: ≥2 interproximal sites with CAL ≥6 mm and ≥1 interproximal site with PPD ≥5 mm.
No periodontitis: none of the above criteria met.

#### EFP/AAP 2018 classification:^52^ diagnosis required

Interdental CAL was present at ≥2 non-adjacent teeth, or
Buccal/oral CAL ≥3 mm with pocketing ≥3 mm was observed at ≥2 teeth, excluding cases attributable to non-periodontal causes (e.g., trauma, caries, endodontic lesions).
Disease severity was staged from I to IV based on CAL, PPD, bone loss, and tooth loss. Extent was categorized as localized (<30% of teeth involved) or generalized (≥30%) criteria.^57^

### Statistical Analysis and Data Visualization

Data were analyzed using STATA/MP version 18.0 (Stata; College Station, TX, USA). Continuous variables were summarized as means and standard errors, and categorical variables as frequencies and percentages. Binary logistic regression was used to assess associations between periodontitis and explanatory variables. Variables with p <0.20 in bivariate analysis were entered into multivariable models, with age and sex retained regardless of significance. Separate multivariable models were constructed for mild-to-moderate periodontitis (stages I–II) and severe periodontitis (stages III–IV), each compared to the absence of periodontitis. Adjusted odds ratios (ORs) and 95% confidence intervals (CIs) were reported.

Geographic map was produced using QGIS version 3.14 (‘Pi’) (QGIS Development Team, 2020), an open-source geographic information system. To visually compare the distribution of periodontitis stages according to both CDC/AAP 2012 and EFP/AAP 2018 classification criteria, a stacked bar chart was created using Microsoft Excel 2019 (Microsoft; Redmond, WA, USA).

### Ethical Approval

The study protocol was reviewed and approved by the National Health Research Ethics Committee of Burkina Faso (Approval No. 2023-02-18; March 2, 2023). Written informed consent was obtained from all participants. Data confidentiality was preserved in accordance with national biomedical research guidelines by assigning anonymized identification numbers for each patient.

## RESULTS

### Sociodemographic and Clinical Characteristics

**Fig 1:**
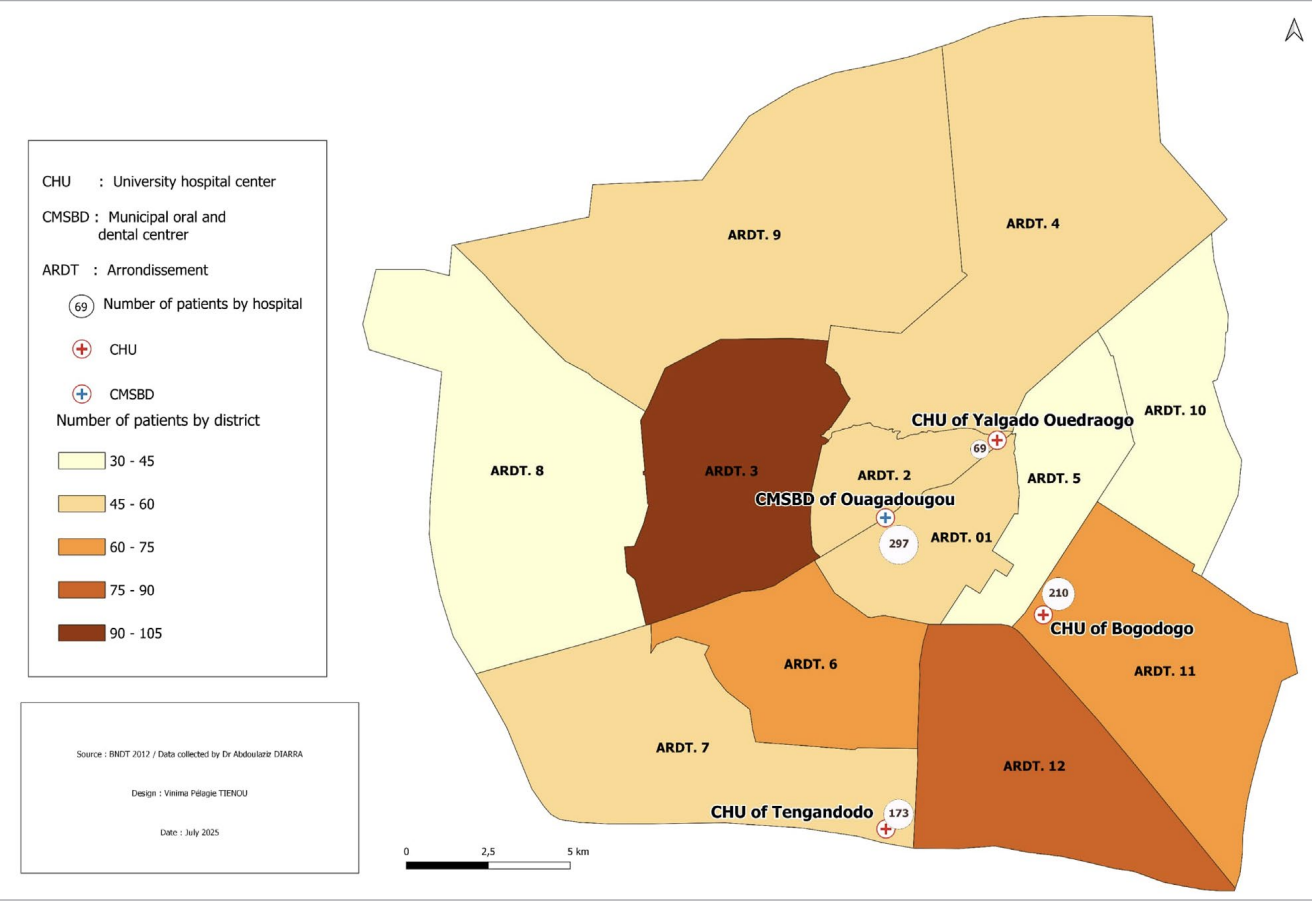
Distribution of participants (number of periodontitis cases) by district (arrondissement) Ouagadougou and sampling site. Districts are color-coded according to the number of cases identified in each area. University Hospital Centers (CHU) and the Municipal Oral and Dental Center (CMSBD) are indicated, with the corresponding number of patients per site. Map designed and produced using GIS software in July 2025.

A total of 749 participants were enrolled, with the Municipal Oral Health Center (CMSBD) contributing the largest proportion (n = 297; 39.65%). Participants were distributed across 12 districts, with district No. 3 being the best represented (Fig 1).

The mean age was 34.32 ± 11.51 years (median: 32), and 52.34% were female (female:male ratio 1.10). Most participants were married (55.94%), had university-level education (46.06%), and were employed in the private sector (71.85%). Only 6.00% reported active health insurance coverage, and 2.27% had diagnosed systemic conditions, predominantly hypertension.

Behavioral risk factors included current smoking in 4.55%, former smoking in 2.14%, and regular alcohol consumption in 7.53%, with an average intake of 2.5 drinks per day. One-quarter (25.00%) had never visited a dentist. Among those who had, 41.07% had attended within the past six months, primarily due to dental pain. More than half (56.00%) had never undergone professional scaling. While 66.22% reported brushing twice or more daily, only 38.85% practiced interdental cleaning, mostly with toothpicks (89.47%). Regular mouthwash use was reported by 8.81% (Table 1 and supplementary Table A1).

**Table 1:** Sociodemographic, health, and behavioral characteristics of study participants (N=749)

Characteristics	n (%)
**Sociodemographic**	
**Residence**	
Urban	678 (90.52)
Peri–urban	69 (9.21)
NR	2 (0.27)
**Age (years)**	34.32 ± 11.51
**Gender**	
Female	392 (52.34)
Male	357 (47.66)
**Marital status**	
Single	319 (42.59)
Married	419 (55.94)
**Educational level**	
Primary	113 (15.09)
Upper secondary	247 (32.98)
University	345 (46.06)
**Professional status**	
Public	208 (27.77)
Private	536 (71.56)
**HEALTH AND BEHAVIOR**	
**Health insurance status**	
No	703 (93.86)
Yes, active	46 (6.14)
**Dental history**	
Ever visited a dentist	560 (74.77)
Tooth loss	320 (42.72)
**Oral hygiene**	
Professional tooth cleaning (ever)	321 (42.86)
Brush teeth once a day	203 (27.10)
Brush teeth twice or more a day	496 (66.22)
Interdental cleaning (yes)	291 (38.85)
**Mouthwash**	
No	653 (87.18)
Yes	66 (8.81)
**Lifestyle habits**	
Smoking status	
Current smoker	34 (4.54)
Former smoker	16 (2.14)
**Alcohol consumption**	
Regularly	56 (7.48)
Occasionally	209 (27.90)
NR: not reported.	

### Prevalence of Periodontitis and Diagnostic Performance of Classifications

Generalized plaque accumulation was observed in 84.07% of participants, with nearly half (49.53%) exhibiting generalized bleeding on probing (BOP). The predominant clinical attachment level (CAL) was 1–2 mm (78.39% ± 17.75), with CAL ≥5 mm found in a minority (2.75% ± 5.67). Shallow pockets (PPD ≤3 mm) predominated (88.66% ± 10.43), while deep pockets ≥6 mm were rare (0.58% ± 2.04) (Table 2).

**Table 2:** Clinical parameters and periodontal diagnosis according to both classification (N=749)

Clinical measurements and diagnosis	Overall (N=749)
**O’Leary Plaque index measures n (%)**	
0%–20%	62 (8.30)
20%–30%	57 (7.63)
30%–100%	628 (84.07)
**BOP measurements, n (%)**	
0%–10%	168 (22.49)
10%–30%	209 (27.98)
30%–100%	370 49.53)
**%CAL 1–2 mm**	
Mean ± SD	78.39 ± 17.75
Median IQR	82.29 22.39–100
**%CAL 3–4 mm**	
Mean ± SD	18.49 ± 14.27
Median IQR	15.59 6.77–28.12
**%CAL ≥ 5 mm**	
Mean ± SD	2.75 ± 5.67
Median IQR	0 0–3.12
**%PPD ≤ 3mm**	
Mean ± SD	88.66 ± 10.43
Median IQR	90.1 84.37–96.87
**%PPD 4 mm**	
Mean ± SD	3.49 ± 4.47
Median IQR	1.56 0–5.72
**%PPD 5 mm**	
Mean ± SD	1.13 ± 2.42
Median IQR	0 0–1.04
**%PPD ≥ 6mm**	
Mean ± SD	0.58 ± 2.04
Median IQR	0 0–0
**Periodontitis prevalence, n (%)**	
According to CDC/AAP 2012	421 (56.21)
According to EFP/AAP 2018	538 (71.83)
BOP: bleeding on probing; CAL: clinical attachment loss; PPD: probing pocket depth. According to the CDC/AAP 2012 method, periodontitis was estimated at 56.21% (95% CI: 52.57–59.80). According to the EFP/AAP method, periodontitis was estimated at 71.83% (95% CI: 68.45–75.03).	

**Fig 2:**
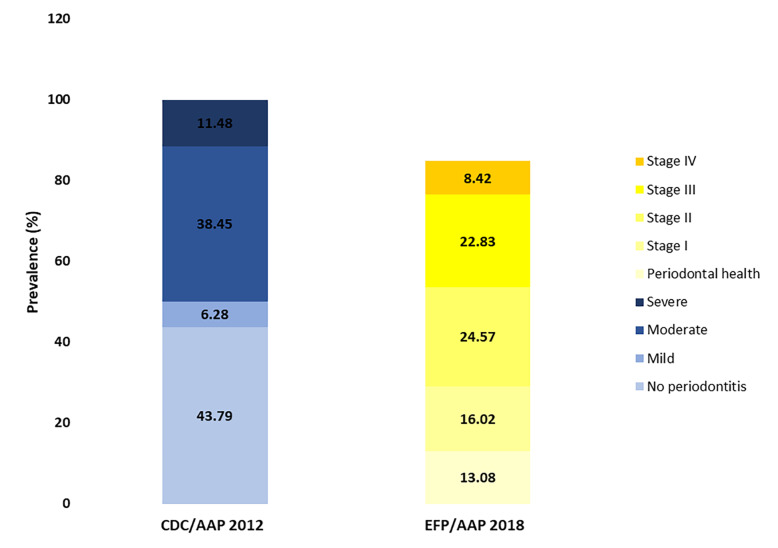
Stacked bar chart showing periodontitis distribution by severity according to CDC/ AAP 2012 and EFP/AAP 2018 Classifications. Prevalence values (%) for each severity category (no periodontitis, mild, moderate, severe for CDC/AAP 2012, periodontal health, Stage I–IV for EFP/AAP 2018) were represented as individual components of the respective bars. This graphical approach allows direct visual comparison of the respective disease burden and the distribution of severity stages across the two classification systems.

Using the CDC/AAP 2012 case definition, periodontitis prevalence was 56.21%, with moderate cases representing the majority (38.45%), followed by severe (11.48%) and mild (6.28%) respectively. The EFP/AAP 2018 classification yielded a higher prevalence of 71.83%, with stage III/IV accounting for 31.25%, stage II for 24.57%, and stage I for 16.02% (Fig 2).

When the CDC/AAP definition was used as the reference standard, the EFP/AAP classification exhibited high sensitivity (99.29%) but low specificity (28.96%). The corresponding positive predictive value (PPV) was 64.21%, and the negative predictive value (NPV) reached 96.94% (supplementary Table A2).

### Risk Factors for Periodontitis

Age was positively associated with periodontitis in both classifications. In univariate analysis, each additional year increased the odds of periodontitis (CDC/AAP 2012: OR = 1.05, 95% CI: 1.04–1.07, p < 0.001; EFP/AAP 2018: OR = 1.05, 95% CI: 1.03–1.08, p < 0.001). This association remained statistically significant after adjustment (CDC/AAP 2012: OR = 1.03, 95% CI: 1.01–1.05, p = 0.001; EFP/AAP 2018: OR = 1.03, 95% CI: 1.00–1.07, p = 0.040).

Tobacco use was linked to a 2.24 to 2.78-fold increased risk (CDC/AAP 2012: OR = 2.78, 95% CI: 1.12–6.89, p = 0.028; EFP/AAP 2018: OR = 2.24, 95% CI: 1.03–4.86, p = 0.04). Other factors associated with higher odds in univariate analysis included being married (OR = 2.88, 95% CI: 2.13–3.89, p < 0.001), although these were not confirmed in multivariate models.

Regular mouthwash use showed a protective association, but was statistically significant only under the EFP/AAP 2018 definition (OR = 0.52, 95% CI: 0.28–0.99, p = 0.045) (Table 3 and supplementary Table A3).

**Table 3:** Univariate and multivariate logistic regression of the risk factors associated with periodontitis according to the two case definitions

Variables	CDC/AAP 2012				EFP/AAP 2018			
	Univariate		Multivariate		Univariate		Multivariate	
	Crude OR (95% CI)	p-value	Adjusted OR (95% CI)	p-value	Crude OR (95% CI)	p-value	Adjusted OR (95% CI)	p-value
Age	1.05 (1.04–1.07)	<0.001	1.03 (1.01–1.05)	0.001	1.05 (1.03–1.08)	<0.001	1.03 (1.00–1.07)	0.040
Marital status								
Never married	1		1		1		1	
Currently married	2.88 (2.13–3.89)	<0.001	1.42 (0.94–2.14)	0.099	2.72 (1.73–4.23)	<0.001	1.28 (0.68–2.42)	0.442
Formerly married	1.68 (0.50–5.61)	0.401	0.73 (0.18–2.99)	0.659	0.80 (0.21–3.13)	0.753	0.26 (0.05–1.35)	0.109
Education level								
No school	1		1		1		1	
Primary school	3.21 (1.53–6.73)	0.002	3.34 (1.48–7.53)	0.004	1.92 (0.51–7.22)	0.332	1.64 (0.34–7.93)	0.537
Secondary school	1.62 (0.85–3.09)	0.143	1.97 (0.95–4.07)	0.067	0.63 (0.21–1.90)	0.416	0.47 (0.13–1.71)	0.255
Tertiary school	0.72 (0.38–1.35)	0.304	0.93 (0.45–1.93)	0.850	0.46 (0.16–1.33)	0.151	0.39 (0.11–1.39)	0.146
Smoking status								
Never smoked	1		1		1		–	
Current smoker	1.31 (0.45–3.83)	0.619	2.78(1.12–6.89	0.028	2.24 (1.03–4.86)	0.042	–	
Former smoker	2.25 (0.29–17.51)	0.439	0.93(0.29–2.98)	0.902	1.04 (0.38–2.81)	0.945	–	
Diabetes								
No	1		–		1		–	
Yes	1.09 (0.13–9.19)	0.934	–		1.04 (0.23–4.68)	0.960	–	
Arterial hypertension								
No	1		–		1		–	
Yes	3.97 (0.86–18.23)	0.077	1.40 (0.28–7.12)	0.683	-		–	
Mouthwash								
No	1		1		1		1	
Yes	0.70 (0.45–1.07)	0.101	0.90 (0.55–1.46)	0.671	0.70 (0.45–1.07)	0.101	0.52 (0.28–0.99)	0.045
CDC/AAP 2012: n= 722, pseudo-R^2^= 0.13; AIC= 898.70, p-value < 0.001. EFP/AAP 2018: n=606, pseudo-R^2^= 0.12; AIC= 483.18, p-value < 0.001.								

### Risk Factors According to Periodontitis Severity

#### Mild-to-moderate periodontitis (stage I–II)

Increasing age remained a statistically significant risk factor (OR = 1.03, 95% CI: 1.01–1.05, p = 0.005). Primary education level was positively associated with mild-to-moderate periodontitis (CDC/AAP 2012: OR = 2.43, 95% CI: 1.02–5.81, p = 0.046). Tobacco use showed a strong association under the CDC/AAP classification (OR = 4.35, 95% CI: 1.58–11.92, p = 0.004). Regular mouthwash use was protective under the EFP/AAP classification (OR = 0.44, 95% CI: 0.23–0.84, p = 0.012) (Table 4 and supplementary Table A4).

**Table 4:** Univariate and multivariate logistic regression of the risk factors associated with mild–moderate and Stage I–II periodontitis

Variables	CDC/AAP 2012				EFP/AAP 2018			
	Mild–moderate periodontitis vs non periodontitis				Stage I–II periodontitis vs non periodontitis			
	Univariate		Multivariate		Univariate		Multivariate	
	Crude OR (95% CI)	p-value	Adjusted OR (95% CI)	p-value	Crude OR (95% CI)	p-value	Adjusted OR (95% CI)	p-value
Age	1.05 (1.03–1.06)	<0.001	1.03 (1.01–1.05)	0.005	1.02 (1–1.05)	0.062	1.02 (0.99–1.06)	0.191
**Gender**								
Male	1		1		1		1	
Female	0.68 (0.50–0.92)	0.014	0.76 (0.53–1.11)	0.153	0.62 (0.39–0.99)	0.044	0.58 (0.35–0.96)	0.035
**Marital status**								
Never married	1		1		1		1	
Currently married	2.67 (1.94–3.66)	<0.001	1.30 (0.83–2.01)	0.249	1.69 (1.06–2.70)	0.028	1.19 (0.63–2.24)	0.594
Formerly married	1 (0.23–4.25)	0.996	0.51 (0.10–2.60)	0.416	0.27 (0.04–1.64)	0.155	0.20 (0.03–1.52)	0.119
**Education level**								
No school	1		1		1			
Primary school	2.8 (1.28–6.13)	0.010	2.43 (1.02–5.81)	0.046	2.16 (0.53–8.82)	0.286		
Secondary school	1.69 (0.85–3.35)	0.133	1.92 (0.89–4.17)	0.100	0.92 (0.28–3.03)	0.895		
Tertiary school	0.77 (0.40–1.51)	0.452	0.89 (0.41–1.95)	0.773	0.84 (0.26–2.68)	0.767		
**Smoking status**								
Never smoked	1		1		1			
Current smoker	2.24 (1.01–5.01)	0.048	4.35 (1.58–11.92)	0.004	1.16 (0.37–3.61)	0.800		
Former smoker	1.01 (0.35–2.91)	0.986	1.24 (0.37–4.24)	0.727	2.32 (0.28–19.08)	0.435		
**Arterial hypertension**								
No	1		1	–	1	–		
Yes	5.02 (1.09–23.07)	0.038	1.90 (0.38–9.61)	0.437	1	–		
Daily fruit consumption	1.29 (1.17–1.42)	<0.001	1.24 (1.12–1.38)	<0.001	1.12 (0.98–1.28)	0.093		
Daily vegetable consumption	1.14(1.05–1.24)	0.001			1.10 (0.97–1.25)	0.150	0.99 (0.84–1.17)	0.931
**Mouthwash**								
No	1		1		1		1	
Yes	0.66 (0.41–1.05)	0.078	0.82 (0.49–1.39)	0.464	0.49 (0.27–0.89)	0.018	0.44 (0.23–0.84)	0.012
CDC/AAP 2012: n= 639, pseudo-R^2^= 0.13; AIC= 813.81, p-value < 0.001. EFP/AAP 2018: n= 388, pseudo-R^2^= 0. 07; AIC= 424.75, p-value= 0.002.								

#### Severe periodontitis (stage III–IV)

Older age was significantly associated with severe periodontitis (CDC/AAP: OR = 1.05, 95% CI: 1.02–1.08, p = 0.001; EFP/AAP 2018: OR = 1.06, 95% CI: 1.02–1.10, p = 0.003). Lower education level was also a significant risk factor (CDC/AAP: OR = 6.02, 95% CI: 1.79–20.20, p = 0.004). Protective factors included tertiary education (EFP/AAP: OR = 0.20, 95% CI: 0.05–0.81, p = 0.024) and employment in the private sector (CDC/AAP: OR = 0.33, 95% CI: 0.18–0.59, p < 0.001). The associations of tobacco use (CDC/AAP definition: OR = 4.98, 95% CI: 1.31–18.98, p < 0.05) and mouthwash (EFP/AAP2018: OR =0.49, 95% CI: 0.26–0.92, p = 0.026) with severe periodontitis observed in univariate analyses were not confirmed in multivariate models (Table 5 and supplementary Table A5).

**Table 5:** Univariate and multivariate logistic regression of the risk factors associated with severe and Stage III–IV periodontitis

Variables	CDC/AAP 2012				EFP/AAP 2018			
	Severe periodontitis vs no periodontitis				Stage III–IV periodontitis vs no periodontitis			
	Univariate		Multivariate		Univariate		Multivariate	
	Crude OR (95% CI)	p-value	Adjusted OR (95% CI)	p-value	Crude OR (95% CI)	p-value	Adjusted OR (95% CI)	p-value
Age	1.07 (1.04–1.09)	<0.001	1.05 (1.02–1.08)	0.001	1.09 (1.06–1.12)	<0.001	1.06 (1.02–1.10)	0.003
**Marital status**								
Never married	1		1		1		1	
Currently married	4.01 (2.33–6.89)	<0.001	1.12 (0.53–2.35)	0.760	5.78 (3.43–9.73)	<0.001	1.59 (0.73–3.46)	0.242
Formerly married	5.31 (1.18–23.84)	0.029	1.49 (0.25–8.90)	0.662	2.43 (0.58–10.22)	0.227	0.24 (0.04–1.51)	0.128
**Education level**								
No school	1		1		1		1	
Primary school	4.70 (1.54–14.34)	0.006	6.02 (1.79–20.20)	0.004	1.79 (0.46–6.94)	0.401	1.27 (0.25–6.43)	0.775
Secondary school	1.37 (0.47–3.96)	0.562	2.35 (0.74–7.50)	0.149	0.46 (0.15–1.45)	0.185	0.36 (0.09–1.48)	0.158
Tertiary school	0.52 (0.18–1.51)	0.231	1.06 (0.33–3.41)	0.918	0.23 (0.74–0.70)	0.010	0.20 (0.05–0.81)	0.024
**Professional status**								
Public employee	1		1		1		1	
Private employee	0.31 (0.19–0.51)	<0.001	0.33 (0.18–0.59)	<0.001	0.57 (0.33–0.97)	0.040	0.53 (0.27–1.05)	0.071
**Smoking status**								
Never smoked	1		1		1			
Current smoker	2.22 (0.72–6.80)	0.164	2.46 (0.62–9.76)	0.199	1.51 (0.49–4.72)	0.475		
Former smoker	1.14 (0.23–5.59)	0.872	1.04(0.15–7.23)	0.967	2.16 (0.25–18.77)	0.484		
**Mouthwash**								
No	1				1		1	
Yes	0.85 (0.42–1.72)	0.649			0.49 (0.26–0.92)	0.026	0.59 (0.26–1.32)	0.198
CDC/AAP 2012: n= 406, pseudo-R2= 0.21; AIC= 349.06, p-value < 0.001. EFP/AAP 2018: n= 322, pseudo-R2= 0.26; AIC= 321.26, p-value < 0.0.								

## DISCUSSION

### Main Findings and Comparison to the Literature

To date, very few epidemiological studies in Africa have adopted up-to-date case definitions for periodontitis such as the CDC/AAP 2012 or the EFP/AAP 2018 classifications.^25,31^ This raises justified concerns regarding the validity and comparability of prevalence data across studies in this region. To our knowledge, this is the first population-based study in sub-Saharan Africa to report periodontitis prevalence using both definitions. Our findings indicate a high prevalence of periodontitis among adults in an urban West African setting. However, estimates varied substantially depending on the case definition used. Notably, the EFP/AAP case definition showed high sensitivity (99.29%) but low specificity (28.96%) relative to the CDC/AAP criteria, suggesting it detects most true cases, but may overestimate prevalence by including individuals with early or mild disease not captured by the CDC/AAP definitions.

These discrepancies are not unexpected given the differences in diagnostic thresholds and clinical criteria, which lead to variations in the number of identified cases.^4,15,29,35^ For example, Costa et al^15^ demonstrated that prevalence varied from 13.8% to 65.3%, depending on the criteria applied to the same dataset.^15^ Notably, the EFP/AAP definition includes any detectable interdental clinical attachment loss (CAL), while the CDC/AAP requires CAL ≥3 mm, and considers only interproximal sites. In addition, the EFP/AAP also considers buccal CAL, increasing its sensitivity for detecting incipient cases.^18,52^ Primarily, designed for clinical practice, the EFP/AAP emphasizes early detection and may overestimate disease burden in population studies. Conversely, the CDC/AAP was developed for epidemiological surveillance, prioritizing specificity and identification of established disease.^44,51^

While variability between case definitions is expected, the magnitude of discrepancy between case definitions depends on disease distribution and severity, as well as the population’s exposure to risk factors. For instance, in rural Southern Brazil, Ortigara et al^51^ reported high agreement between the CDC/AAP and EFP/AAP definitions in a population with predominantly severe periodontitis (71% Stage III/IV). A similar pattern was observed in another Brazilian study focusing exclusively on severe periodontitis, with nearly identical estimates (CDC/AAP: 47.3%; EFP/AAP stage III/IV: 43.2%).^8^

In contrast, a study from Turkey reported 100% prevalence under EFP/AAP versus 61.9% using CDC/AAP, with more than twice the number of severe cases (34% vs 16.8%).^23^ Similarly, Morales et al^44^ reported substantial discrepancies among adolescents across five South American countries: 75.6% prevalence using EFP/AAP versus 27.2% with CDC/AAP. In adults, the EFP/AAP demonstrated lower specificity and poor discrimination (AUC = 0.57, 95% CI: 0.53–0.62) when compared to CDC/AAP as the reference, consistent with our findings. This likely reflects the reduced diagnostic accuracy of case definitions in populations with higher disease prevalence.^56^ Furthermore, in populations with predominantly severe periodontitis, prevalence estimates from both definitions tend to converge, while greater divergence is observed when mild to moderate cases predominate. This highlights the importance of reporting prevalence using multiple case definitions to improve the robustness and comparability of epidemiological data, particularly in understudied regions.^59^

Using the CDC/AAP 2012 definition, mild periodontitis accounted for only 6.28% of cases in our relatively young sample (mean age 34.32 ± 11.51 years). In contrast, Kocher et al^33^ reported 54.2–69.1% “no or mild” periodontitis among German adults aged >40 years who regularly implemented preventive care (80–90% brushing ≥2×/day; ≥1 dental visit/year), underscoring the influence of lifelong exposure to preventive services. Nevertheless, the prevalence of severe periodontitis was more comparable across populations (11.48% in our study vs 6.3–10.7% in Kocher et al^33^), supporting the notion that interindividual susceptibility and rapid disease progression may play a role irrespective of healthcare access.^36^

Having ≥20 teeth is essential for maintaining adequate oral function and overall health. Lang et al^36^ recently summarized longitudinal data on the natural history of periodontal disease and identified a mean CAL of 1.81 mm at age 30 years as a threshold predicting retention of ≥20 teeth at age 60. In our relatively young sample (mean age 34.32 ± 11.51 years), more than 20% already exhibited mean CAL >2 mm, suggesting that a substantial proportion may be at increased risk of losing functional dentition over time.

In line with previous literature, increasing age (3% per year), smoking (OR 2.24–2.78), and lower education levels were consistently associated with higher periodontitis risk, regardless of the case definition used.^17,19,53^ However, the strength and statistical significance of associations with other risk or protective factors were influenced by the case definition, particularly in relation to disease severity. These observations are consistent with prior studies demonstrating that differences in case definitions can statistically significantly alter observed associations with risk factors, including those involving systemic conditions such as glycemic control and adverse pregnancy outcomes. This variability likely reflects differences in the number of cases identified under each classification.^29,35,39^

An interesting finding was the inverse association between regular mouthwash use and periodontitis. While antimicrobial rinses such as chlorhexidine have well-documented anti-plaque and anti-gingivitis effects, evidence supporting their preventive role in periodontitis remains limited in epidemiological research.^41^ In our adjusted models, the association was statistically significant only for stage I/II periodontitis under the EFP/AAP classification. This raises the possibility of gingivitis misclassification as early-stage periodontitis due to the heightened sensitivity of this case definition. Furthermore, considering the potential alterations to the oral microbiome caused by over-the-counter mouthwashes, their routine use should be confined to specific indications until stronger evidence supports their recommendation as a population-level preventive measure against periodontitis.^9^

### Strengths and Limitations

A major strength of this study is the application of both CDC/AAP 2012 and EFP/AAP 2018 case definitions, providing a more comprehensive assessment of disease prevalence and facilitating cross-study comparisons. Additional strengths include the large sample size, the broad geographic representation across Ouagadougou, and the full-mouth six-site examinations performed by a limited number of trained examiners. Nonetheless, several limitations should be acknowledged. The cross-sectional design limits causal inference regarding the influence of lifestyle or behavioral factors. Furthermore, complete staging and grading could not be performed due to missing information on the cause of tooth loss, HbA1c values, and radiographic data. Although examiners were trained by the principal investigator, no formal intra- or inter-examiner calibration was performed, which may introduce measurement variability.

Although participants represented diverse sociodemographic backgrounds and districts, the lack of random sampling may limit the generalizability of our findings to the broader population. Additionally, the hospital-based setting may have introduced selection bias, potentially overrepresenting individuals with more severe disease or those with greater awareness and financial resources to seek oral care. Whether patients attending the four major public dental centres in Ouagadougou are fully representative of the city’s adult population is a legitimate concern. Nonetheless, these centers constitute the only public dental facilities in the city, offer high patient turnover, and provide the standardized clinical infrastructure necessary for full-mouth periodontal examinations, which made them the most feasible sites for this investigation. Our results should therefore be interpreted in light of these methodological constraints and may not be entirely generalizable. Future studies should adopt probabilistic sampling strategies to enhance representativeness at the population level. Nonetheless, our estimates reflect similar patterns to a recent study from the Greater Accra region of Ghana, another West African capital, where random stratified sampling was employed (CDC/AAP 46.7% periodontitis, 13.9% severe cases), lending support to the validity of our findings.^25^

### Implications for Practice and Research

The high prevalence of periodontitis (56.21–71.83%) and the notable burden of severe disease (11.5–31.4%) among a relatively young (mean age 34.32 ± 11.51 years), well-educated urban population with access to public dental infrastructure is a cause for concern. These findings call for urgent public health interventions aimed at improving oral hygiene awareness—especially in light of widespread generalized plaque accumulation (84.1%)—and promoting education on modifiable risk factors, including tobacco use.

Notably, earlier national data from rural Burkina Faso (2014) reported higher levels of CAL ≥4 mm among adolescents and adults (21% and 61%, respectively), suggesting potential urban–rural disparities in periodontal health.^13^ Further research is needed to determine whether these differences reflect true epidemiological patterns. Longitudinal studies are also warranted to explore how urbanization and rural-to-urban migration impact the risk and progression of periodontitis in African populations.

## CONCLUSION

Periodontitis affects over half of this urban West African adult population, with severe disease present in up to one-third. Moderate agreement between EFP/AAP and CDC/AAP case definitions suggests reduced accuracy of the former in high-prevalence settings. Age and smoking were consistently associated with periodontitis risk, whereas associations with other factors varied by case definition.

## ACKNOWLEDGEMENTS

The authors gratefully acknowledge the support of the hospital directors and staff who facilitated data collection. This study was supported by the National Fund for Research and Innovation for Development (FONRID), Project AAP3/MalaInfect/NCP/PC/2021.

### Artificial intelligence (AI) use statement

No generative or non-generative AI-assisted technologies were used in the writing, analysis, editing, or production of this manuscript. All text, data analysis, tables, and figures were produced entirely by the authors without assistance from AI tools.

## APPENDIX

**Table A1:** Additional characteristics of study participants (N=749)

Characteristics	n (%)
Sociodemographic	
*Ethnicity* Mossi Pular Gourmantché Gourounsi Bissa Others NR	506 (67.74) 13 (1.74) 21 (2.81) 22 (2.95) 38 (5.09) 147 (19.68) 2 (0.27)
*Religion* Muslim Christian Others	352 (47.00) 390 (52.07) 6 (0.93)
*Health insurance status * No Yes, active NR	702 (93.72) 46 (6.14) 1 (0.13)
General health information	
*Medical history * No Yes NR	649 (86.65) 99 (13.22) 1 (0.13)
*Comorbidities* No Yes Don’t know Diabetes HTA Arthritis Depression Chronic kidney failure Cardiovascular diseases Cataract	717 (95.72) 17 (2.27) 15 (2.01) 7 (0.93) 12 (1.60) 1 (0.13) 1 (0.13) 1 (0.13) 2 (0.27) 1 (0.13)
*Current medication * No Yes	657 (87.72) 92 (12.28)
*Dental attendance * Once a year Twice or more/year Only if needed	51/560 (9.10) 13/560 (2.32) 496/560 (88.58)
*Time of last visit * Within the last six months Within the last year Less than 5 years ago More than 5 years ago Long ago, I don’t remember Other reasons	230 (41.07) 143 (25.54) 111 (19.82) 27 (4.82) 39 (6.96) 10 (1.79)
*Main reason for last visit* Pain Gum problems Tooth mobility Tooth decay Routine check–up Mouth sores Other	383 (68.39) 20 (3.57) 2 (0.36) 76 (13.57) 22 (3.93) 1 (0.18) 56 (10.00)
*Tooth loss * No Yes	429 (57.28) 320 (42.72)
Oral hygiene	
*Time of last professional tooth cleaning * Within the last six months Within the last year Less than 5 years ago More than 5 years ago Long ago, I don’t remember	97 (29.57) 113 (34.45) 67 (20.43) 11 (3.35) 40 (12.20)
*Main cleaning tool * Toothbrush	748 (99.87)
*Main complementary cleaning tool * No Yes Dental floss Interdental brush Chew sticks Toothpicks	388 (51.80) 361 (48.20) 30 (8.31) 4 (1.11) 4 (1.11) 323 (89.47)
*Knowledge of interdental brushes * No/don’t know Yes NR	670 (89.46) 72 (9.61) 7 (0.93)
*Interdental brushe use * No Yes NR	724 (97.71) 17 (2.29) 8 (1.07)
*Tongue cleaning* No Yes Sometimes NR	131 (17.49) 582 (77.70) 35 (4.67) 1 (0.13)
Lifestyle habits	
*Smoking status* Non-smoker Current smoker Former smoker NR	697 (93.06) 34 (4.54) 16 (2.14) 2 (0.27)
*Alcohol consumption* Never Regularly Occasionally Formerly NR	445 (59.41) 56 (7.48) 209 (27.90) 34 (4.54) 5 (0.67)
*Weekly number of glasses * Mean ± SD Median [IQR]	2.49 ± 2.97 1 [1–2]
*Daily fruit consumption * Mean ± SD Median [IQR]	1.85 ± 1.86 1 [1–2]
*Weekly fruit consumption* Mean ± SD Median [IQR]	3.44 ± 2.46 0
*Daily vegetable consumption* Mean ± SD Median [IQR]	2.71 ± 2.27 2 [1–4]
*Weekly vegetable consumption* Mean ± SD Median [IQR]	4.23 ± 2.24 5 [2–6]
	

**Table A3:** Additional univariate and multivariate logistic regression of the factors associated with periodontitis according to the two case definitions

Variables	CDC/AAP 2012				EFP/AAP 2018			
	Univariate		Multivariate		Univariate		Multivariate	
	Crude OR (95% CI)	p-value	Adjusted OR (95% CI)	p-value	Crude OR (95% CI)	p-value	Adjusted OR (95% CI)	p-value
*Residence*								
Urban	1				1			
Rural	0.84 (0.51–1.37)	0.477	–		0.80 (0.40–1.59)	0.518	–	
*Religion*								
Muslim	1		1		1		–	
Christian	0.78 (0.58–1.05)	0.096	0.90 (0.64–1.28)	0.556	0.79 (0.51–1.22)	0.289	–	–
Other	0.27 (0.05–1.41)	0.121	0.34 (0.06–2.05)	0.241	0.40 (0.07–2.11)	0.277	–	–
*Alcohol consumption*			–				–	
Never	1		–		1		–	
Drink regularly	0.56 (0.27–1.17)	0.122	–		1.24 (0.70–2.20)	0.459	–	
Occasional drinking	0.66 (0.41–1.07)	0.093	–		0.88 (0.63–1.22)	0.437	–	
Former drinker	0.47 (0.18–1.23)	0.122	–		0.66 (0.33–1.33)	0.248	–	
*Genetic family antecedents*								
No	1		1		1		1	
Yes	0.80 (0.41–1.55)	0.706	1.22 (0.67–2.20)	0.516	1.10 (0.67–1.82)	0.706	0.87 (0.40– 1.90)	0.719
Don’t know	2.08 (1.64–3.07)	<0.001	–		2.24 (1.64–3.07)	<0.001	–	
*Family dental antecedents*								
No	1		1		1		1	
Yes	1.11(0.58–2.13)	0.749	0.76(0.44–1.32)	0.325	1.16 (0.73–1.84)	0.537	0.98 (0.45–2.10)	0.952
Don’t know	2.27(1.39–3.70)	0.001			2.49 (1.81–3.43)	<0.001		
*Daily fruit consumption*	1.17 (1.02–1.35)	0.023	1.21 (1.09–1.34)	<0.001	1.29 (1.18–1.41)	<0.001		
*Weekly fruit consumption*	1.18 (1.07–1.31)	0.001			1.10 (1.03–1.17)	0.003	1.15 (1.01–1.30)	0.036
*Daily vegetable consumption*	1.13(1.00–1.28)	0.058	–		1.12 (1.04–1.21)	0.003	0.99 (0.85–1.16)	0.890
Weekly vegetable consumption	1.01 (0.91–1.11)	0.885	1.04 (0.97–1.13)	0.283	1.05 (0.99–1.12)	0.115	–	
*Professional cleaning*								
No	1		1		1		1	
Yes	1.45 (0.93–2.26)	0.101	1.04 (0.72–1.49)	0.836	1.41 (1.05–1.89)	0.023	1.27 (0.75–2.14)	0.368
CDC/AAP 2012: n= 722, pseudo-R2= 0.13; AIC= 898.70, p-value < 0.001. EFP/AAP 2018: n=606, pseudo-R2= 0.12; AIC= 483.18, p-value < 0.001.								

**Table A2:** Diagnostic performance of CDC/AAP 2012 versus EFP/AAP 2018 criteria

Indicator	Value (%)	95%CI
Sensitivity	99.29	[98.69%–99.89%]
Specificity	28.96	[25.71%–32.21%]
Positive predictive value	64.21	[60.78%–67.64%]
Negative predictive value	96.94	[95.71%–98.17%]
Prevalence (reference)	56.21	[52.66%–59.76%]
Results are percentages with 95% confidence intervals (95%CI). Analyses with STATA/MP 18.0, N = 749. The CDC/AAP 2012 definition was used as the reference standard.		

**Table A4:** Additional univariate and multivariate logistic regression of the risk factors associated with mild–moderate and Stage I–II periodontitis

Variables	CDC/AAP 2012				EFP/AAP 2018			
	Mild–moderate periodontitis vs non periodontitis				Stage I–II periodontitis vs non periodontitis			
	Univariate		Multivariate		Univariate		Multivariate	
	Crude OR (95% CI)	p-value	Adjusted OR (95% CI)	p-value	Crude OR (95% CI)	p-value	Adjusted OR (95% CI)	p-value
*Residence*								
Urban	1				1			
Rural	0.75 (0.44–1.29)	0.299			0.84 (0.40–1.75)	0.635		
*Religion*								
Muslim	1		1		1			
Christian	0.81 (0.59–1.10)	0.175	1 (0.68–1.46)	0.996	0.87 (0.55–1.39)	0.569		
Other	0.35 (0.07–1.81)	0.209	0.68 (0.10–4.80)	0.699	0.29 (0.04–2.16)	0.229		
*Professional status*								
Public employee	1		1		1			
Private employee	0.71 (0.50–1.01)	0.055	0.64 (0.43–0.97)	0.035	0.77 (0.45–1.30)	0.326		
*Tobacco*								
No	1				1			
Yes	7.44 (2.58–21.45)	<0.001			6.42 (0.85–48.61)	0.072		
*Alcohol consumption*								
Never	1		1		1		1	
Drink regularly	1.25 (0.69–2.27)	0.469	0.64 (0.29–1.38)	0.252	0.47 (0.21–1.05)	0.064	0.41 (0.17–1.00)	0.050
Occasional drinking	0.91 (0.64–1.28)	0.580	1.04 (0.70–1.57)	0.837	0.78 (0.47–1.30)	0.349	0.77 (0.45–1.31)	0.330
Former drinker	0.47 (0.21–1.07)	0.071	0.32 (0.13–0.82)	0.017	0.40 (0.14–1.19)	0.101	0.35 (0.11–1.11)	0.075
Weekly number of glasses	1.04 (0.98–1.10)	0.202			0.95 (0.89–1.02)	0.128		
*Genetic family antecedents*								
No	1				1		1	
Yes	0.72 (0.43–1.20)	0.207			0.63 (0.32–1.25)	0.189	0.76 (0.37– 1.60)	0.475
*Family dental antecedents*								
No	1		1		1			
Yes	0.73(0.45–1.17)	0.193	0.71(0.41–1.23)	0.222	0.68 (0.35–1.34)	0.266		
*Diabetes*								
No	1				1			
Yes	1.31 (0.29–5.90)	0.726			0.32 (0.02–5.17)	0.422		
*Weekly fruit consumption*	1.12 (1.05–1.20)	0.001			1.14 (1.02–1.26)	0.016	1.14 (1.00–1.31)	0.047
*Weekly vegetable consumption*	1.07 (1–1.14)	0.054	1.07 (0.99–1.16)	0.104	0.98 (0.89–1.09)	0.766		
*Professional cleaning*								
No	1		1		1			
Yes	1.57 (1.15–2.14)	0.004	1.10 (0.76–1.61)	0.605	1.21 (0.76–1.93)	0.427		
CDC/AAP 2012: n= 639, pseudo-R^2^= 0.13; AIC= 813.81, p-value < 0.001. EFP/AAP 2018: n= 388, pseudo-R^2^= 0. 07; AIC= 424.75, p-value= 0.00.Table S5 Additional univariate and multivariate logistic regression of the risk factors associated with severe and stage III–IV periodontitis								

**Table A5:** Additional univariate and multivariate logistic regression of the risk factors associated with severe and stage III–IV periodontitis

Variables	CDC/AAP 2012				EFP/AAP 2018			
	Severe periodontitis vs no periodontitis				Stage III–IV periodontitis vs no periodontitis			
	Univariate		Multivariate		Univariate		Multivariate	
	Crude OR (95% CI)	p-value	Adjusted OR (95% CI)	p-value	Crude OR (95% CI)	p-value	Adjusted OR (95% CI)	p-value
*Residence*								
Urban	1				1			
Rural	1.17 (0.55–2.48)	0.678			0.74 (0.34–1.61)	0.45		
*Religion*								
Muslim	1				1			
Christian	0.68 (0.42–1.10)	0.116			0.69 (0.43–1.11)	0.129		
Other	1				0.51 (0.08–3.18)	0.473		
*Tobacco*								
No	1				1			
Yes	4.98 (1.31–18.98)	0.019			6.64 (0.86–50.96)	0.069		
*Alcohol consumption*								
Never	1				1		1	
Drink regularly	1.22 (0.49–3.02)	0.671			0.68 (0.31–1.49)	0.335	3.34 (0.12–0.99)	0.048
Occasional drinking	0.76 (0.43–1.35)	0.349			0.51 (0.30–0.88)	0.016	0.51 (0.26–1.02)	0.056
Former drinker	1.42 (0.56–3.58)	0.457			0.54 (0.19–1.57)	0.257	0.72 (0.18–2.89)	0.640
Weekly number of glasses	1.03 (0.96–1.11)	0.417			0.95 (0.89–1.02)	0.177		
*Genetic family antecedents*								
No	1				1		1	
Yes	0.92 (0.43–1.99)	0.830			0.5 (0.24–1.05)	0.067	0.62 (0.24– 1.57)	0.310
*Family dental antecedents*								
No	1				1			
Yes	0.95(0.47–1.92)	0.879			0.92 (0.46–1.81)	0.801		
*Diabetes*								
No	1				1			
Yes	1				2.12 (0.24–18.37)	0.496		
*Arterial hypertension*								
No	1				1			
Yes	1				1			
*Daily fruit consumption*	1.24 (1.08–1.43)	0.003	1.16 (0.97–1.37)	0.103	1.28 (1.08–1.51)	0.005		
*Weekly fruit consumption*	1.02 (0.93–1.12)	0.669			1.25 (1.12–1.40)	<0.001	1.23 (1.05–1.44)	0.009
*Daily vegetable consumption*	1.02(0.93–1.12)	0.635			1.17 (1.02–1.35)	0.026	1.09 (0.90–1.33)	0.378
Weekly vegetable consumption	0.99 (0.89–1.10)	0.881			1.03 (0.93–1.15)	0.517		
*Professional cleaning*								
No	1				1		1	
Yes	0.90 (0.55–1.48)	0.682			1.83 (1.13–2.96)	0.014	1.14 (0.59–2.20)	0.699
CDC/AAP 2012: n= 406, pseudo-R2= 0.21; AIC= 349.06, p-value < 0.001. EFP/AAP 2018: n= 322, pseudo-R2= 0.26; AIC= 321.26, p-value < 0.00.								
